# The relationship between post-traumatic sleep and related symptoms in children with high-energy trauma: a study based on ecological momentary assessment

**DOI:** 10.3389/fped.2026.1754778

**Published:** 2026-07-15

**Authors:** Xiaoyan Feng, Xuelian Zhu, Lihong Zhu, Yanhong Ding, Yunfei Wei, Bijun Hui, Xuefang Wu

**Affiliations:** Affiliated Children’s Hospital of Jiangnan University, Wuxi, Jiangsu, China

**Keywords:** children, ecological momentary assessment, high-energy trauma, sleep, symptoms

## Abstract

**Introduction:**

High-energy trauma is characterized by severe injuries, complex clinical conditions, and high mortality rates. Trauma exposure itself induces stress and excessive alertness, leading to sleep disturbances, which in turn exacerbate anxiety, depression, and the risk of post-traumatic stress disorder, while delaying natural recovery after trauma. However, screening for sleep disorders in hospitalized children with high-energy trauma remain insufficient. This study aimed to explore early post‑traumatic sleep status and its associations with related symptoms in children with high‑energy trauma using ecological momentary assessment (EMA).

**Method:**

A longitudinal observational design was adopted. Children with high‑energy trauma admitted to our hospital from July 1, 2024, to May 15, 2025 were recruited using convenience sampling. Sleep status and related symptoms were continuously monitored for the first five days post‑trauma. Data were analyzed using generalized estimating equations (GEE) and multivariate linear regression.

**Results:**

A total of 69 patients were included in the final analysis. Pain was the most severe symptom, followed by anxiety, fear, and fatigue. GEE revealed that pain, anxiety, fear, and dizziness were significantly associated with sleep quality (*P* < 0.001, *P* = 0.033, *P* = 0.012, *P* = 0.034); pain, itching, and numbness were significantly associated with reduced sleep efficiency (*P* < 0.001, *P* = 0.003, *P* = 0.004), whereas fatigue was significantly associated with improved sleep efficiency (*P* = 0.032); pain and fear were significantly associated with nocturnal awakenings (*P* < 0.001, *P* = 0.009). Moreover, symptoms were often inter‑related. Multivariate linear regression showed that factors affecting sleep quality included pain on day 1 and day 3 (*P* = 0.001, *P* = 0.018), fear on day 4 (*P* = 0.045), and fear and itching on day 5 (*P* = 0.011, *P* = 0.017). Regarding sleep efficiency, the primary influencing factor were: pain on day 1 post-trauma (*P* = 0.001), numbness at the affected site on day 2 (*P* = 0.004), fear, pruritus, and numbness at the affected site on day 4 (*P* = 0.034, *P* < 0.001, *P* < 0.001), and pruritus on day 5 (*P* < 0.001).

**Discussion:**

Children with high‑energy trauma exhibit certain sleep disturbances in the early post‑traumatic period, and the main factors influencing sleep change over time. Close attention should be paid to sleep disorders and their associated influencing factors, and appropriate interventions should be implemented.

## 1 Introduction

In recent times, the incidence of high-energy traumas has increased. High-energy trauma refers to severe trauma caused by the transfer of substantial kinetic energy into the body through mechanisms such as falls from heights, traffic accidents, crush injuries, industrial accidents, and war weapons (1). These traumas are characterized by intense force, severe damage, and complex conditions, causing both physical and psychological harm (2, 3). Research indicates that sleep disorders such as insomnia, early awakening, frequent awakenings, and trauma-related nightmares are typical behavioral responses following trauma (4, 5). Early recognition and management of sleep disturbance in post-trauma patients, particularly those at risk, can improve their functional and emotional outcomes (6–8). Expert consensus (9) highlights that pain, sleep, and anxiety interact with one another, and addressing these issues lays the foundation for enhancing patient satisfaction and accelerating recovery. However, current research pays insufficient attention to early hospitalization sleep monitoring in children with high-energy injuries, as well as to the correlation between sleep patterns and symptoms in such cases. The ecological momentary assessment (EMA) method provides repeated, real-time evaluations of an individual's daily cognition, emotions, environment, and behaviors, reducing recall bias compared with traditional assessment methods (10). Against this background, this study aims to explore the correlation and trends of sleep status and related symptoms in children with high-energy injury during hospitalization through the EMA of sleep and related symptoms to provide a practical basis for the formulation of precise interventions and nursing plans for sleep in children with high-energy trauma.

Trauma is a leading cause of the global disease burden (11) and presents significant challenges for trauma care (12). High-energy injuries impose a substantial burden on both health and economy (13, 14). Sleep is a fundamental element of health and wellbeing that affects both physiological processes and quality of life (6). Insomnia and trauma-related nightmares are the most common symptoms, and these nightmares are often severe and distressing (15). In patients with early-stage trauma, nightmares, frequent awakening, and trauma-related nightmares significantly impair sleep quality (5). Sleep disorders remain a prevalent yet frequently overlooked complaint among trauma survivors. Current research on high-energy injuries in pediatric patients primarily focuses on basic studies, epidemiological surveys, prehospital emergency care, and treatment approaches. Most existing studies have addressed sleep issues in patients with post-traumatic stress disorder (PTSD), traumatic brain injury (TBI), and spinal cord injury or in children admitted to the pediatric intensive care unit (PICU). However, comprehensive research on sleep patterns in populations with high-energy injuries is scarce. Symptom assessment still predominantly relies on questionnaire surveys, which involve the retrospective collection of symptom data from specific time periods and subsequent completion of questionnaires. However, this method is prone to recall bias, leading to imprecise clinical guidance for symptom management. Recently, scholars have proposed innovative assessment techniques such as EMA. During disease progression and treatment, symptoms are not static but continuously evolve over time. Scholars emphasize the importance of temporal considerations in symptom perception and management (16, 17). Sleep diary monitoring based on momentary ecological assessments has become common. Guided by this model, herein, we investigate the correlation and temporal patterns of sleep-related symptoms in pediatric patients with high-energy trauma during hospitalization to provide recommendations for precision nursing care in this clinical context.

## 2 Methods

### 2.1 Patients

Convenience sampling was used to select pediatric patients with high-energy injuries admitted to the hospital from 1 July 2024 to 15 May 2025 who met the inclusion and exclusion criteria.

The inclusion criteria were as follows: (1) 5 ≤ age < 18 years old; (2) injuries resulting from high-energy trauma mechanisms (18, 19), including traffic accident injuries and falls from heights, as determined by physician evaluation; (3) injuries occurring within 24 h of admission; (4) being conscious and able to respond appropriately; (5) obtaining informed consent from caregivers.

The exclusion criteria were as follows: (1) hospitalization duration of less than 5 days; (2) condition remained unstable; (3) presence of other severe chronic diseases; (4) pre-existing neurological or psychiatric disorders; (5) pre-existing sleep disorder; (6) presence of medical disputes.

The discontinuation criteria were as follows: (1) withdrawal during the study period; (2) death or disease progression preventing completion of valid assessment; (3) premature discharge for various reasons.

The sample size was calculated using the one-way repeated-measures method in PASS 2025 software, referencing the values of the primary outcome measure (sleep quality) at the first five time points from Jia et al. (20). The statistical test employed the Greenhouse–Geisser-corrected *F*-test, with the effect size multiplier K set to 1. Assuming equal correlations among all measurements, a correlation coefficient of 0.2 between adjacent time points, and 90% statistical power, the required sample size was given as 43 subjects (five measurements per subject). The study was approved by the hospital's ethics committee (WXCH2024-05089).

### 2.2 Research tools

#### 2.2.1 General data survey form

The survey form was developed by the researcher based on reference materials, including age, sex, only-child status, place of residence, primary caregiver, admitting department, cause of injury, injury type, main injured body parts, polytrauma status, surgical history, emergency treatment experience, and analgesic use.

#### 2.2.2 Chinese version of the 10-item big five personality inventory (TIPI-C)

The TIPI-C comprises 10 items across five factors (21). Specifically, it investigates Emotional Stability in children through an emotional stability factor that includes two items. This factor has been shown to have a Cronbach's *α* of 0.62 and a criterion-referenced validity of 0.58. The scale employs a Likert 7-point scale where 1 indicates “strongly disagree” and 7 indicates “strongly agree.” Higher scores indicate better emotional stability.

### 2.3 Monitoring tools for sleep status and related symptoms

#### 2.3.1 Objective sleep assessment

This section utilizes an event-based evaluation method developed in the EMA research. After a child's sleep cycle ends, the child or caregiver must document sleep details. According to the international consensus on sleep diaries (22), daily sleep logs should include bedtime, sleep onset time, wake-up time, number of awakenings during sleep, and self-rated sleep quality. In this study, the child recorded these observations based on subjective feelings or with the help of caregivers. The self-rating scale ranges from 0 to 10 points, where 0 indicates the worst sleep quality and 10 represents the best. Higher scores indicate better sleep quality. Sleep efficiency was calculated as actual sleep duration/(wake-up time-bedtime preparation time) × 100%.

#### 2.3.2 EMA daily symptom recording

This section describes the design, using time- and event-based assessment methods from the EMA research framework. Through a literature review and synthesis, three time periods were selected for routine evaluation: upon waking, at 14:00, and before bedtime to assess post-traumatic sleep-related symptoms in children with high-energy injuries. Through literature review and brainstorming, the most common sleep-related symptoms in these children were identified as pain, anxiety, fear, gastrointestinal discomfort, fatigue, itching, numbness at the site of injury, and dizziness. The Changhai Pain Scale was used to assess pain in children aged 8 years. The visual analog scale (VAS) was employed to interpret the numerical rating scales developed by Chinese scholars using a 0–10 scale with textual descriptions (23). The revised facial expressions scale (FES) was used for children under 8 years of age. This scale features six realistic facial expressions paired with numerical ratings of 0, 2, 4, 6, 8, and 10 (24). Itching and fatigue were assessed using a Likert scale with 10-point severity ratings (0–10), where 0 indicates no symptoms and 10 represents the most severe symptoms (25, 26). All assessments demonstrated good reliability and validity. Anxiety, fear, gastrointestinal discomfort, numbness at the injury site, and dizziness were evaluated by using scoring methods adapted from those used to assess itching and fatigue.

#### 2.3.3 Body movement recorder

This device was developed in recent years for monitoring activity intensity, has been widely adopted in the field of sleep medicine, and has been extensively used in pediatrics (27). In this study, the Xiaomi Mi Band 2 was selected as the activity recorder. Following the product instructions, the device was worn on a child's wrist for dynamic sleep monitoring. Key sleep parameters included total sleep duration, deep sleep, light sleep, waking frequency, and sleep quality. The activity recorder demonstrated good reliability and validity for sleep monitoring, with a strong correlation with polysomnography (PSG). The American Academy of Sleep Medicine emphasizes actigraphy as an effective tool for sleep research, particularly in conditions such as circadian rhythm disorders and insomnia (28). The monitoring data served as a reference for objective sleep assessment in pediatric patients admitted to the PICU and in those willing to use the device.

### 2.4 Data collection methods

#### 2.4.1 Collection method

A friendly nurse–patient relationship was established with the caregivers of the enrolled children. The survey content was presented in a concise cartoon booklet to create a sleep diary. This booklet explains the purpose and significance of the study to the children and their families, emphasizes the objectives and methodology of using the sleep diary, and clarifies that multiple questionnaires will be administered during the study to obtain the support and cooperation of the children and their families. The body movement recorder adhered to the principle of voluntariness, and for pediatric patients admitted to the PICU, the researchers provided detailed instructions on proper wearing techniques and precautions. The recorder was used from 20:00 on the day of administration until 08:00 the following morning. On the first day, one-on-one on-site guidance was provided for questionnaire completion. Daily reviews were conducted to identify and correct errors and incomplete entries promptly. Objective sleep assessments and EMA daily symptom records were documented in paper format. Each morning, the assigned nurse conducted an inquiry, combining data from the body movement recorder (if available) or records provided by the family regarding the child's sleep patterns the previous night; subsequent shifts of nurses or family members completed the symptom evaluation forms. For sleep quality scores and other symptom ratings, children under 8 years of age were primarily assessed by their families based on the child's self-reported symptoms and behaviors, while children aged 8 and older were primarily evaluated by the children themselves.

#### 2.4.2 Data collection timing

After admission, when the child's condition stabilized, both the general information survey form and the Big Five Personality Inventory were administered. Given the rapid bed turnover in trauma care and the need to assess early post-traumatic sleep patterns, continuous monitoring was conducted for 5 days following injury. Objective sleep assessment covered the period from admission stabilization with clear consciousness until day 5, with daily morning evaluations of the previous night's sleep lasting for five consecutive days. Symptom documentation involved three time periods each day (morning, 14:00, and bedtime) and was monitored for five consecutive days in parallel with sleep tracking.

Compliance enhancement small gifts were provided at the time of admission to improve compliance. After caregivers completed the corresponding sleep and symptom assessments, stickers and small gifts were given to the children. At the same time, they were informed that any problems during hospitalization could be raised.

### 2.5 Statistical methods

A statistical analysis was performed using SPSS 26.0 software. For continuous variables, those conforming to a normal distribution were described by means of mean ± standard deviation, while those not conforming to this distribution were expressed as median and interquartile range; categorical variables were presented as frequencies and percentages. Line graphs and bar charts were used to illustrate the trends in sleep and symptom changes. For longitudinal data with a normal distribution and homogeneity of variance, one-way repeated-measures ANOVA was employed; for data lacking a normal distribution or homogeneity of variance, generalized estimating equations (GEE) were applied. Pairwise rank-sum tests were performed for between-group comparisons. GEEs were used to analyze the associations between sleep and symptoms as well as among the symptoms themselves, and multiple linear regression was employed to identify factors influencing daily sleep quality or efficiency. Statistical significance was set at a level of *P* < 0.05.

## 3 Results

### 3.1 General information

The sample size of this study was 75 cases, and 6 cases were excluded (due to early discharge or withdrawal), resulting in a final sample size of 69 cases (see Table 1).

**Table 1:** General information of children (*n* = 69).

Item	Category	Number of cases (%)
Gender	Boy	47 (68.12)
	Girl	22 (31.88)
The only child	Yes	35 (50.72)
	No	34 (49.28)
Domicile	City or town	54 (78.26)
	Country	15 (21.74)
Residence department	Orthopedics	37 (53.62)
	Neurosurgery	20 (28.99)
	Burn and plastic surgery	4 (5.80)
	PICU	3 (4.35)
	General surgery department	3 (4.35)
	Department of stomatology	2 (2.90)
Primary carers	Mother	41 (59.42)
	Father	20 (28.99)
	Grandparent	4 (5.80)
	Nurse	3 (4.35)
	Others	1 (1.45)
Time from injury to admission	＜1 h	6 (8.70)
	1–2 h	13 (18.84)
	2–4 h	35 (50.72)
	＞4 h	15 (21.74)
Causes of injury	Traffic accident	65 (94.20)
	Falling from a height	4 (5.80)
Main injury site	Limb	25 (36.23)
	Head	20 (28.99)
	Chest/abdomen	4 (5.80)
	Spine/pelvis	6 (8.70)
	Others	5 (7.25)
	Merging two or more	9 (13.04)
Type of injury	Closed injury	48 (69.57)
	Open injury	21 (30.43)
Multiple injury	Yes	12 (17.39)
	No	57 (82.61)
Being rescued	Yes	7 (10.14)
	No	62 (89.86)
Surgery	Yes	17 (24.64)
	No	52 (65.36)
Using painkillers	Yes	19 (27.54)
	No	50 (72.46)
Age (year, $\bar{X}+S$)		9.22 ± 3.17

### 3.2 Sleep status and sleep symptom scores of patients

**Figure 1:**
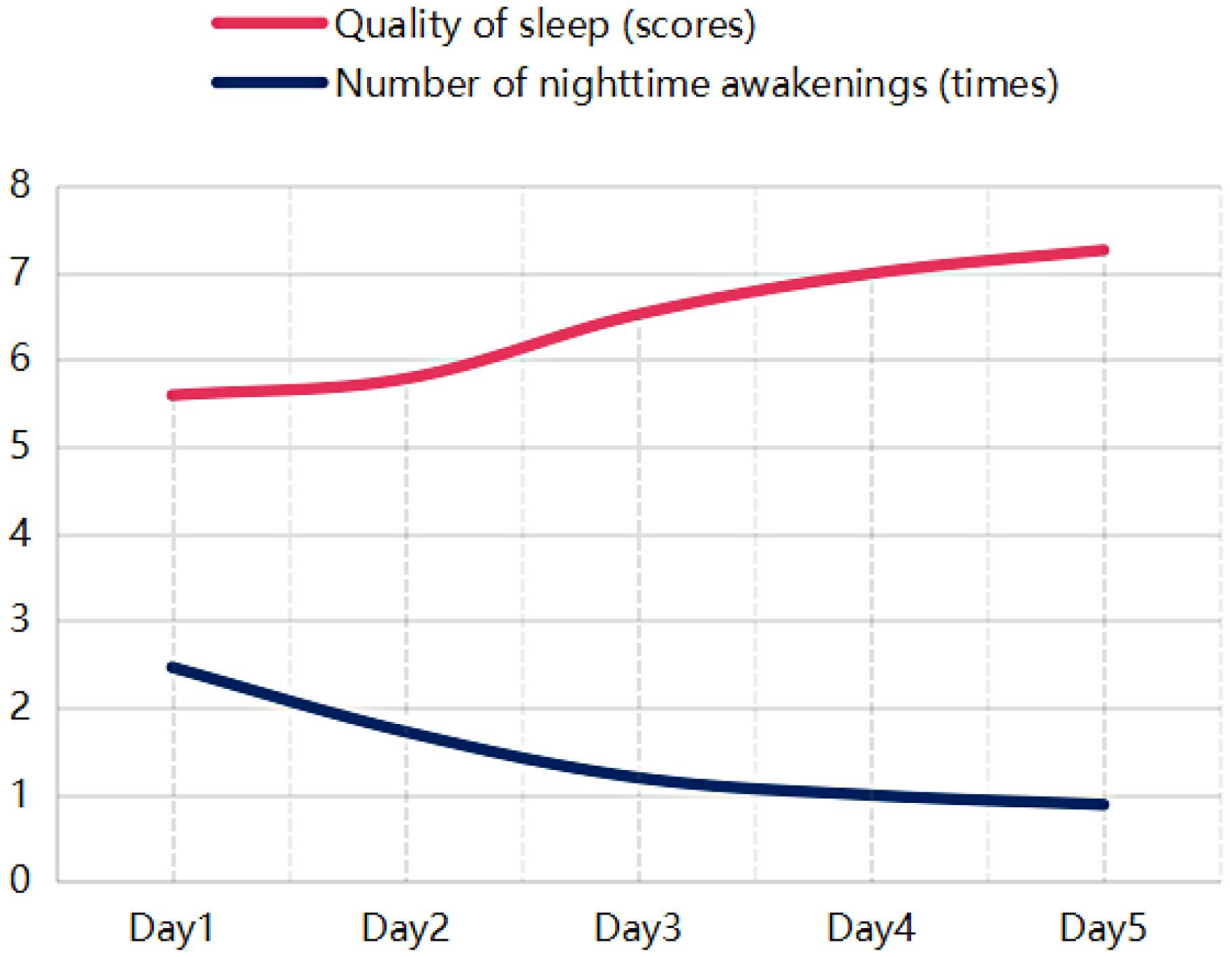
Sleep patterns 5 days after trauma.

**Figure 2:**
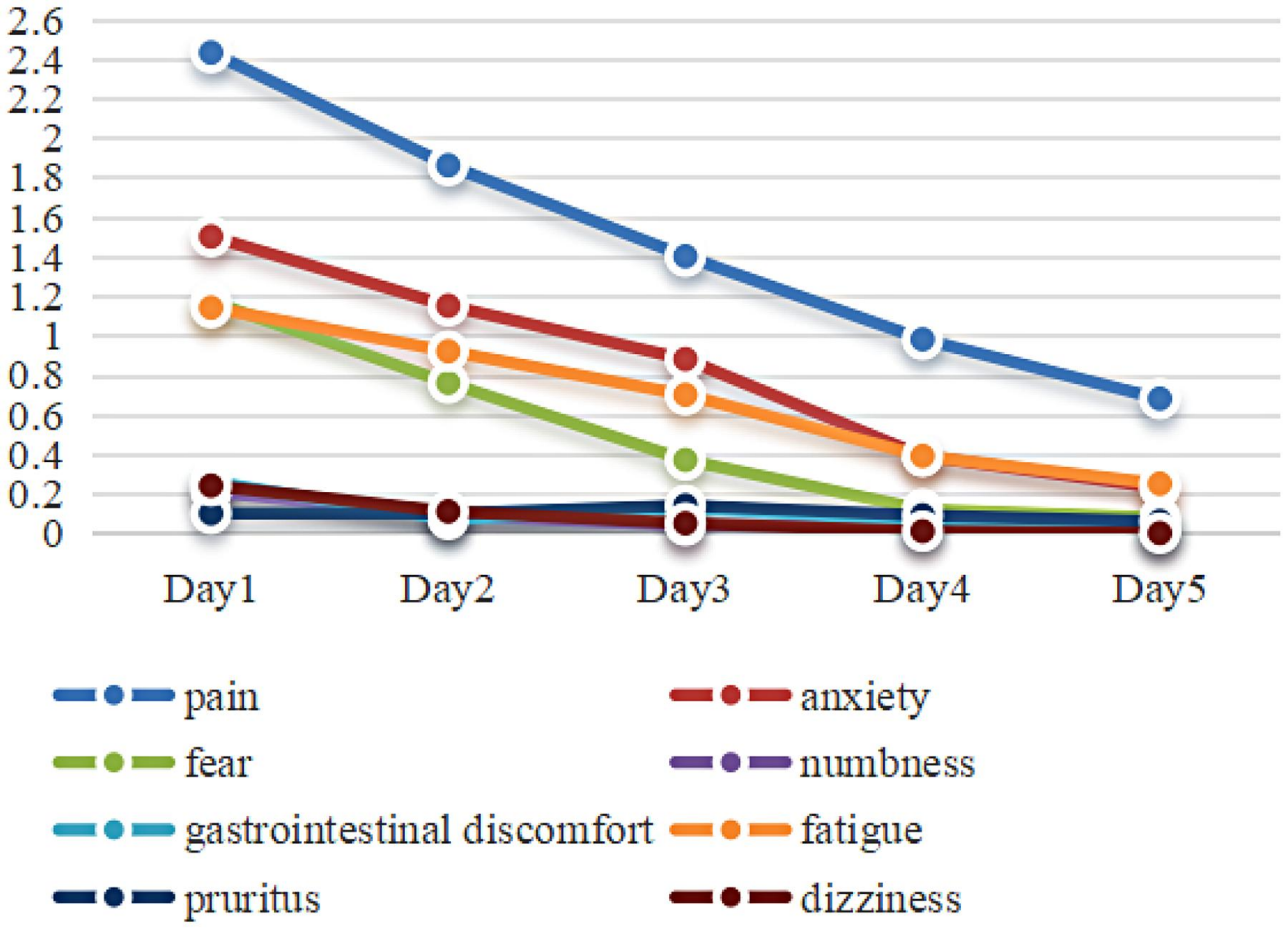
Post-traumatic sleep-related symptom scores at 5 days.

**Figure 3:**
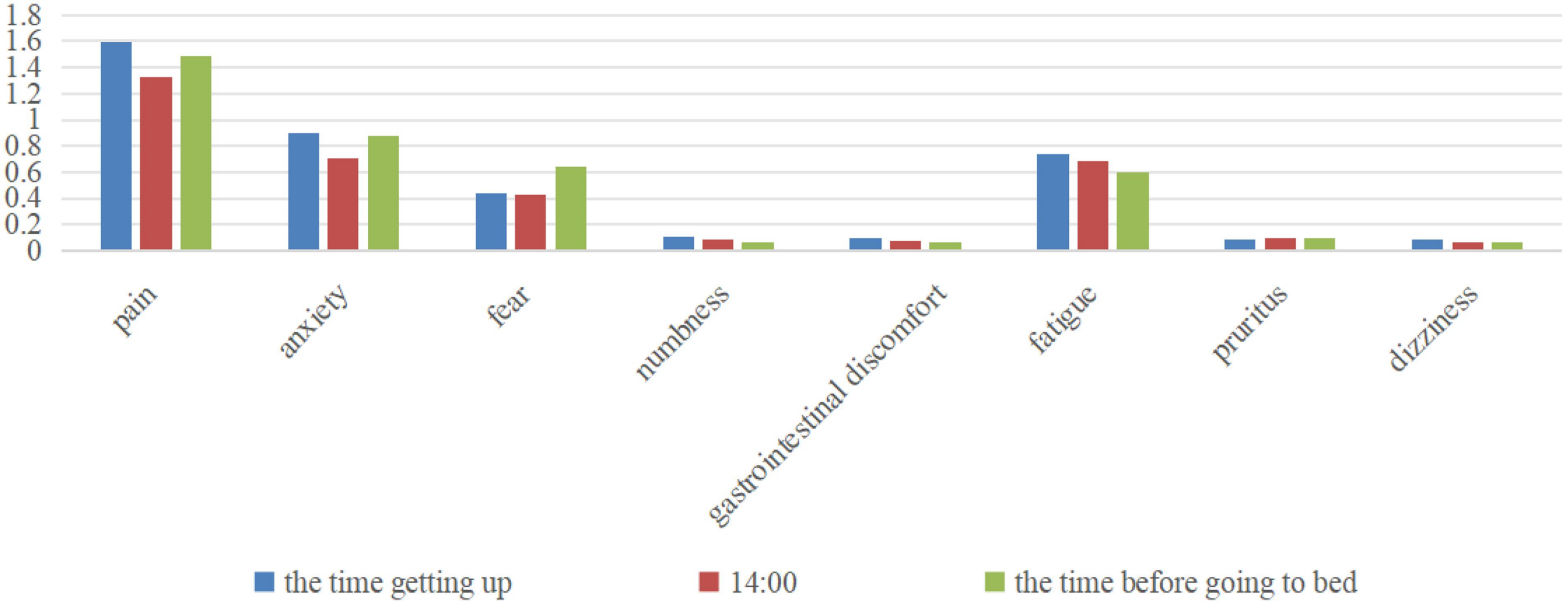
Severity of sleep symptoms at different time periods during the first 5 days after trauma.

On the first post-trauma day, sleep efficiency and quality were generally low, with frequent nighttime awakenings. On the second day, the sleep quality scores showed no significant improvement, although statistically significant differences remained. By the fourth day, neither sleep efficiency nor nighttime awakenings showed a marked improvement compared with the previous day. Both sleep quality and sleep efficiency demonstrated gradual improvement trends, with nighttime awakenings progressively decreasing (Table 2, Figure 1). Pain was the primary cause of nighttime awakening in patients, accounting for 23.19% of cases involving nightmares (Table 3). Pain was the most severe symptom, and pain, anxiety, and fatigue persisted for relatively long periods (Figure 2). The most severe symptoms upon waking included pain, anxiety, numbness at the injury site, gastrointestinal discomfort, fatigue, and dizziness. Severe itching occurred around 14:00, and fear was more pronounced before bedtime (Figure 3).

**Table 2:** Sleep status and sleep-related symptoms 5 days after trauma.

Item	Day 1	Day 2	Day 3	Day 4	Day 5	Wald *χ*^2^	*P*
Sleep quality {scores, [M (P_25_, P_75_)]}	6 (4, 7)	6 (5, 7)	6 (5.5, 8)^a^	7 (6, 8)^a^	8 (6, 9)	47.215	<0.001
Sleep Efficiency {rate, [M (P_25_, P_75_)]}	0.89 (0.77, 0.95）	0.91 (0.83, 0.95)^a^	0.94 (0.90, 0.97)^a^	0.94 (0.91, 0.96)	0.96 (0.93, 0.98)	32.974	<0.001
Number of nights awake {sequence, [M (P_25_, P_75_)]}	3 (1, 3)	2 (1, 2)^a^	1 (0, 2)^a^	1 (0, 1)	1 (0, 1)	102.769	<0.001

a Indicates a statistically significant difference compared with the previous day.

**Table 3:** Wake-up events and nightmare situations at night (*n* = 69).

Item	n
Reasons to wake-up	
Pain	158 (38.63)
Anxiety	66 (16.14)
Fear	50 (12.22)
Gastrointestinal discomfort	12 (2.93)
Light/sound	33 (8.07)
Limitation of movement	40 (9.78)
Dizziness	6 (1.47)
Others	40 (9.78)
Pruritus	4 (0.98)
Number of children experiencing nightmares/night terror	16 (23.19)
Number of children experiencing nightmares/night terror exceeding twice	8 (50.00)

### 3.3 Correlation between sleep quality, sleep efficiency, nighttime wakefulness frequency, and sleep symptoms

The correlations among the primary sleep indicators are given in Table 4. Using the factors of sleep efficiency, sleep quality, and nighttime wakefulness frequency and various symptoms as dependent variables, generalized estimation equations were formulated to analyze the correlations between these factors. The meaningful variables are listed in Table 5, and the complete table is provided in Supplementary Tables S5-1 and S5-2. After a Bonferroni correction for multiple comparisons (corrected *α* = 0.05/55 ≈ 0.0009), the generalized estimation equation analysis revealed that pain was significantly associated with reduced sleep quality (OR = 0.668), reduced sleep efficiency (OR = 0.966), and increased nighttime awakenings (OR = 1.302). With regard to correlations among symptoms, anxiety (OR = 1.261) and numbness in the affected area (OR = 1.713) were significantly associated with pain; pain (OR = 1.248) and fear (OR = 1.538) were significantly associated with anxiety; and anxiety (OR = 1.404) was significantly associated with fear. Multivariate linear regression models were employed to examine the relationship between early post-traumatic sleep symptoms, sleep quality, and sleep efficiency in pediatric patients (Tables 6, 7). The meaningful variables are listed in Tables 6 and 7, and the complete table is provided in Supplementary Tables S6, 7. It was revealed that the primary factors influencing sleep quality on post-traumatic day 1 and day 3 were pain (*P* = 0.001 and *P* = 0.018), while fear (*P* = 0.045) was the dominant factor on day 4. On day 5, both fear (*P* = 0.011) and pruritus (*P* = 0.017) were significant contributors. In terms of sleep efficiency, the primary influencing factors were pain (*P* = 0.001) on day 1 after trauma, numbness at the affected site (*P* = 0.004) on day 2, fear (*P* = 0.034), pruritus (*P* < 0.001), and numbness at the affected site (*P* < 0.001) on day 4, and pruritus (*P* < 0.001) on day 5.

**Table 4:** Correlation among the primary sleep indicators.

Item	Sleep quality	Sleep efficiency	Number of nighttime awakenings
Sleep quality	—	0.520	−0.578
Sleep efficiency	0.520	—	−0.605
Number of nighttime awakenings	−0.578	−0.605	—

**Table 5:** Correlation matrix of generalized estimation equations between sleep status and sleep-related symptoms 5 days after trauma in children.

Item	Statistical value	Sleep quality	Sleep efficiency	Number of nighttime awakenings	Pain	Anxiety	Fear	Gastrointestinal discomfort	Fatigue
Pain	OR	0.668^a^	0.966^a^	1.302^a^	—	1.248^a^	1.127^b^	1.048^b^	1.120
Anxiety	OR	0.809^b^	1.000	1.084	1.261^a^	—	1.404^a^	1.031	1.128
Fear	OR	0.781^b^	0.974	1.250^b^	1.124	1.538^a^	—	0.958	1.090
Gastrointestinal discomfort	OR	1.144	0.991	1.141	1.287^a^	1.124	0.867	—	1.063
Fatigue	OR	1.022	1.013^b^	1.035	1.118^b^	1.188^b^	1.075	0.990	—
Pruritus	OR	0.904	0.953^b^	1.1	1.069	0.944	0.965	1.022	0.739^b^
Numbness	OR	0.859	0.937^b^	1.06	1.713^a^	0.84	1.142	1.032	0.992
Dizziness	OR	0.710^a^	1.010	1.219	1.010	0.872	1.138	1.160	1.285

OR, odds ratio. The emotional stability personality traits of the children were analyzed as covariates. Multiple comparisons were corrected using the Bonferroni method, with a corrected significance level of *α* = 0.05/55 ≈ 0.0009.

a Indicates that the corrected *P*-value remains <0.0009.

b Indicates an uncorrected *P*-value <0.05.

**Table 6:** Correlation between sleep quality and symptoms 5 days after trauma in children (*n* = 69).

Time	Variable	Regression coefficient	Standard error	Standardized regression coefficient	*t*	*P*
Day 1	Pain	−0.948	0.267	−0.471	−3.547	0.001
Day 3	Pain	−0.533	0.220	−0.330	−2.427	0.018
Day 4	Fear	−1.396	0.683	−0.254	−2.043	0.045
Day 5	Fear	−2.103	0.806	−0.297	−2.610	0.011
	Pruritus	−1.520	0.621	−0.280	−2.448	0.017

**Table 7:** Correlation between sleep efficiency and symptoms 5 days after trauma (*n* = 69).

Time	Variable	Regression coefficient	Standard error	Standardized regression coefficient	*t*	*P*
Day 1	Pain	−0.105	0.029	−0.466	−3.674	0.001
Day 2	Numbness	−0.096	0.032	−0.36	−3.037	0.004
Day 4	Fear	−0.047	0.022	−0.140	−2.175	0.034
	Pruritus	−0.158	0.012	−0.794	−13.483	<0.001
	Numbness	−0.191	0.044	−0.416	−4.347	<0.001
Day 5	Pruritus	−0.179	0.024	−0.695	−7.343	<0.001

## 4 Discussion

### 4.1 Early post-traumatic sleep dynamics in children with high-energy trauma

This study revealed significant sleep disturbances in children with high-energy trauma during the initial hospitalization period, consistent with the findings of Yuan et al. (29). On the first post-trauma day, both sleep efficiency and quality were notably compromised, accompanied by frequent nighttime awakenings. Trauma may sensitize the arousal center of the central nervous system, disrupting normal sleep–wake regulatory mechanisms (30). Previous studies have linked traumatic events to childhood sleep disorders such as nightmares and insomnia (31). Over time, this study observed gradual improvements in sleep quality and efficiency, along with reduced nighttime awakenings, likely reflecting diminishing acute stress responses, the effects of pain management interventions, and environmental adaptation. However, sleep problems persisted on the fifth day after the trauma. Notably, the process of sleep improvement showed a nonlinear trend: sleep quality scores stagnated on the second day, and sleep efficiency and awakening frequency showed no significant change on the fourth day, indicating that there was a “plateau” in post-traumatic sleep recovery. Pain accounted for most nighttime awakenings, with 23.19% of patients experiencing nightmares or night terrors during the study period, demonstrating the psychological stress caused by high-energy injuries. However, this rate was lower than that reported by Wamser-Nanney and Chesher (32), possibly because of the inclusion of patients treated in the general department. Recurring nightmares can trigger sleep phobia, potentially leading to the development of maladaptive sleep patterns that exacerbate nightmare-induced traumatic memories (33). Traumatic sleep disturbance also has time-specific effects on PTSD symptoms (21). Therefore, clinicians should prioritize monitoring sleep issues during the early post-traumatic phases and conduct thorough nighttime screenings to prevent the progression of PTSD.

### 4.2 Early post-traumatic sleep symptoms and their correlations in high-energy-trauma children

This study revealed that pain is the most severe symptom in the early post-trauma period, followed by anxiety, fear, and fatigue. These three symptoms—pain, anxiety, and fatigue— persist for relatively longer durations. Pain, anxiety, fear, fatigue, and dizziness scores peaked on the first day and showed a significant downward trend thereafter. Anxiety scores declined gradually during the first three days but dropped notably on the fourth day. Itching symptoms peaked on day. Morning-onset symptoms include pain, anxiety, numbness at the site of injury, gastrointestinal discomfort, fatigue, and dizziness. Itching was most severe at noon, whereas fear was most pronounced at bedtime. Interventions should focus on the following corresponding time points: morning pain, numbness, and dizziness correlate with the morning surge in inflammatory mediators and positional changes; itching peaks in the afternoon because of increased histamine release (34) resulting from elevated daytime body temperature; and fear intensifies before sleep. Therefore, a circadian rhythm–based intervention approach should be implemented to enhance pain management in the morning, improve skincare in the afternoon, and provide fear exposure therapy before bedtime. After a Bonferroni correction, a significant bidirectional positive correlation was observed between pain and anxiety, as well as between pain and numbness at the affected site. A significant bidirectional positive correlation also existed between anxiety and fear. These findings indicate that symptoms in children during the acute phase of high-energy trauma are not isolated; rather, they interact through physiological and psychological mechanisms, collectively exacerbating sleep disturbances (9). Clinical care should move beyond a single-symptom management approach and effectively intervene to halt the cycle of symptom interaction and progression.

### 4.3 Dynamic correlation between early post-traumatic sleep symptoms and sleep status in children with high-energy trauma

The results of generalized estimation equation analysis showed that within 5 days after the trauma, pain was significantly associated with reduced sleep quality, decreased sleep efficiency, and nocturnal awakenings. Pain can disrupt sleep through activation of the sympathetic nervous system and metabolic alterations. Given the existence of a shared regulatory network between pain and sleep, effective pain management is crucial (35, 36). Ecological momentary assessments by Jia et al. (20) revealed that postoperative sleep quality in orthopedic patients correlated with anxiety and depressive symptoms within 1 week and nighttime awakening was associated with pain, fatigue, nausea, and vomiting, although this finding differs slightly from the results of our study, likely because of differences in study subjects and timing. The results of the multiple linear regression analysis indicated that on days 1 and 3 post-trauma, sleep quality and efficiency were primarily influenced by pain, which was likely directly related to the intense pain caused by acute trauma. On days 4 and 5, fear significantly impacted sleep quality and efficiency, while numbness at the injury site notably affected sleep efficiency on days 2 and 4. Traumatic experiences typically enhance cognitive and physical arousal, particularly during the presleep phase when nightmares exacerbate phobic symptoms (37). Therefore, early targeted interventions should focus on children exhibiting fear-related symptoms, especially nightmares, to mitigate subsequent fear-induced sleep disturbances. By day 5 post-trauma, itching became the primary factor affecting sleep quality, and on days 4 and 5, itching dominated sleep efficiency. This finding suggests that itching may gradually become a significant factor in recovery. Studies have also found that patients with itching experience sleep deprivation (38), which likely stems from inflammatory mediators, such as histamine, released by mast cells that play crucial roles in skin wound healing (39). Therefore, the key factors influencing sleep patterns vary across different post-trauma periods, indicating that intervention strategies should be adjusted according to the specific recovery phase. For instance, pain relief should be prioritized during the initial post-trauma phase, whereas attention to the effects of fear, itching, and numbness becomes more critical after the acute phase.

## 5 Conclusion

Children with high-energy trauma exhibit specific sleep disturbances during the early post-traumatic phase. Sleep management should prioritize pain control, while addressing psychological symptoms and other contributing factors by employing personalized multidisciplinary strategies. The findings of this study can guide stage-based precision interventions for patients with high-energy trauma. However, the limitations of the study include the short (5-day) hospitalization period, traditional paper-based documentation, and limited sample size. Future studies should expand the sample size, extend the observation duration, use information technology to implement EMA, add wearable devices as conditions allow further exploration of the development of trauma-related sleep disorders and their impact on physical function, and carry out ecological momentary interventions to refine sleep management protocols to enhance the physical and psychological recovery of children.

## Data Availability

The original contributions presented in the study are included in the article/Supplementary Material; further inquiries can be directed to the corresponding authors.
