# Supplementary material for: The relationship between post-traumatic sleep and related symptoms in children with high-energy trauma: a study based on ecological momentary assessment
Source: Front Pediatr. 2026 Jul 15;14:1754778. doi: 10.3389/fped.2026.1754778 (PMC13415361; doi:10.3389/fped.2026.1754778)

Supplementary Material

Note: Table 5 (concise version) in the main text has been split into Table 5-1 and Table 5-2 (detailed versions) below.

Tables

Table 1 General information of children (n=69)

Item		Number of cases（%）
Gender	boy	47（68.12）
	girl	22（31.88）
The only child	yes	35（50.72）
	no	34（49.28）
Comicile	city or towns	54（78.26）
	country	15（21.74）
Residence department	orthopedics	37（53.62）
	neurosurgery	20（28.99）
	burn and plastic surgery	4（5.80）
	PICU	3（4.35）
	general surgery department	3（4.35）
	department of stomatology	2（2.90）
Primary carers	mother	41（59.42）
	father	20（28.99）
	grandparent	4（5.80）
	nurse	3（4.35）
	others	1（1.45）
Time from injury to admission	＜1h	6（8.70）
	1-2h	13（18.84）
	2-4h	35（50.72）
	＞4h	15（21.74）
Causes of injury	traffic accident	65（94.20）
	falling from a height	4（5.80）
Main injury site	limb	25（36.23）
	head	20（28.99）
	chest/abdomen	4（5.80）
	spine/pelvis	6（8.70）
	others	5（7.25）
	merging two or more	9（13.04）
Type of injury	closed injury	48（69.57）
	open injury	21（30.43）

Multiple injury	yes	12（17.39）
	no	57（82.61）
Being rescued	yes	7（10.14）
	no	62（89.86）
Surgery	yes	17（24.64）
	no	52（65.36）
Using painkillers	yes	19（27.54）
	no	50（72.46）
Age(year，$\bar{x}+s$)		9.22±3.17

Table 2: Sleep status and sleep related symptoms 5 days after trauma

Item	Day 1	Day 2	Day 3	Day 4	Day 5	Wald χ^2	*P*
Sleep quality (scores,[M(P_{25},P_{75})])	6(4,7)	6(5,7)	6(5.5,8)*	7(6,8)*	8(6,9)	47.215	＜0.001
Sleep Efficiency (rate,[M(P_{25},P_{75})])	0.89(0.77,0.95）	0.91(0.83, 0.95)*	0.94(0.90, 0.97)*	0.94(0.91, 0.96)	0.96(0.93, 0.98)	32.974	＜0.001
Number of nights awake（sequence,[M(P_{25},P_{75})]）	3（1,3）	2（1,2）*	1(0,2)*	1(0,1)	1(0,1)	102.769	＜0.001

Note:* indicates a statistically significant difference compared with the previous day.

Table 3: Wake-up events and nightmare situations at night (n=69)

Item	n
Reasons to wake-up	
pain	158（38.63）
anxiety	66（16.14）
fear	50（12.22）
gastrointestinal discomfort	12（2.93）
light/sound	33（8.07）
limitation of movement	40（9.78）
dizziness	6（1.47）
others	40（9.78）
pruritus	4（0.98）
number of children experiencing nightmares/night terror	16（23.19）
number of children experiencing nightmares/night terror exceeding twice	8（50.00）

Table 4: Correlation among the primary sleep indicators

Item	Sleep quality	Sleep efficiency	Number of nighttime awakenings
Sleep quality	-	0.520	-0.578
Sleep efficiency	0.520	-	-0.605
Number of nighttime awakenings	-0.578	-0.605	-

Table 5-1: Correlation matrix of generalized estimation equation between sleep status and sleep related symptoms 5 days after trauma in children

Item		Sleep quality	Sleep efficiency	Number of nighttime awakenings
Pain	*r*	-0.403	-0.035	0.264
	OR	0.668	0.966	1.302
	P	＜0.001	＜0.001	＜0.001
Anxiety	*r*	-0.212	0	0.081
	OR	0.809	1	1.084
	P	0.033	0.97	0.41
Fear	*r*	-0.247	-0.026	0.223
	OR	0.781	0.974	1.25
	P	0.012	0.101	0.009
Gastrointestinal discomfort	*r*	0.134	-0.009	0.132
	OR	1.144	0.991	1.141
	P	0.596	0.415	0.376
Fatigue	*r*	0.022	0.013	0.034
	OR	1.022	1.013	1.035
	P	0.785	0.032	0.639
Pruritus	*r*	-0.101	-0.048	0.096
	OR	0.904	0.953	1.1
	P	0.361	0.003	0.509
Numbness	*r*	-0.152	-0.065	0.058
	OR	0.859	0.937	1.06
	P	0.511	0.004	0.803
Dizziness	*r*	-0.343	0.01	0.198
	OR	0.71	1.01	1.219
	P	0.034	0.161	0.261

Note: The emotional stability personality traits of the children were analyzed as covariates. R refers to the coefficient, OR refers to the odds ratio, and *P* refers to the p-value.

Table 5-2: Correlation matrix of generalized estimation equation between sleep status and sleep related symptoms 5 days after trauma in children

Item	Pain	Anxiety	Fear	Gastrointestina	Fatigue	Pruritus	Numbness	Dizziness

					l discomfort				
	r	-	0.221	0.119	0.047	0.114	0.041	0.113	-0.004
Pain	OR	-	1.248	1.127	1.048	1.12	1.041	1.12	0.996
	P	-	＜0.001	0.026	0.042	0.058	0.226	0.065	0.829
	r	0.232	-	0.340	0.031	0.12	-0.014	-0.047	-0.028
Anxiety	OR	1.261	-	1.404	1.031	1.128	0.986	0.954	0.973
	P	＜0.001	-	＜0.001	0.244	0.114	0.323	0.303	0.321
	r	0.538	0.431	-	-0.043	0.086	-0.017	0.043	0.03
Fear	OR	1.713	1.538	-	0.958	1.09	0.983	1.044	1.03
	P	＜0.001	＜0.001	-	0.078	0.302	0.175	0.332	0.399
	r	0.253	0.117	-0.142	-	0.061	0.081	0.037	0.128
Gastrointestinal discomfort	OR	1.287	1.124	0.867	-	1.063	1.085	1.044	1.137
	P	0.029	0.264	0.056	-	0.475	0.326	0.332	0.315
	r	0.112	0.173	0.072	-0.01	-	-0.063	-0.007	0.056
Fatigue	OR	1.118	1.188	1.075	0.99	-	0.939	0.993	1.058
	P	0.039	0.016	0.248	0.657	-	0.057	0.728	0.112
	r	0.067	-0.058	-0.036	0.022	-0.303	-	-0.016	-0.032
Pruritus	OR	1.069	0.944	0.965	1.022	0.739	-	0.984	0.968
	P	0.746	0.255	0.35	0.634	0.025	-	0.483	0.234
	r	0.538	-0.174	0.133	0.031	-0.008	-0.027	-	-0.028
Numbness	OR	1.713	0.84	1.142	1.032	0.992	0.974	-	0.972
	P	＜0.001	0.239	0.292	0.579	0.926	0.235	-	0.225
	r	0.01	-0.137	0.129	0.149	0.251	-0.064	-0.039	-
Dizziness	OR	1.01	0.872	1.138	1.16	1.285	0.938	0.962	-
	P	0.928	0.211	0.296	0.261	0.054	0.338	0.144	-

Note: The emotional stability personality traits of the children were analyzed as covariates. R refers to the coefficient, OR refers to the odds ratio, and *P* refers to the p-value.

Table 6: Correlation between sleep quality and symptoms 5 days after trauma in children（n=69）

Time	Variable	Regression coefficient	Standard error	Standardized regression coefficient	*t*	*P*
Day 1	pain	-0.948	0.267	-0.471	-3.547	0.001
	anxiety	-0.201	0.168	-0.161	-1.193	0.238
	fear	-0.087	0.175	-0.067	-0.498	0.620
	gastrointestinal discomfort	-0.634	0.420	-0.171	-1.511	0.136
	fatigue	0.168	0.136	0.146	1.239	0.220
	pruritus	-0.055	0.356	-0.018	-0.155	0.877
	numbness	-0.047	0.251	-0.021	-0.186	0.853
	dizziness	-0.329	0.297	-0.129	-1.108	0.272
Day 2	pain	-0.444	0.243	-0.247	-1.829	0.072
	anxiety	0.173	0.192	0.133	0.901	0.371

	fear	-0.104	0.253	-0.057	-0.411	0.683
	gastrointestinal discomfort	0.435	0.758	0.069	0.574	0.568
	fatigue	-0.103	0.156	-0.088	-0.659	0.513
	pruritus	0.402	0.409	0.120	0.984	0.329
	numbness	-0.746	0.592	-0.155	-1.261	0.212
	dizziness	-0.605	0.542	-0.142	-1.118	0.268
Day 3	pain	-0.533	0.220	-0.330	-2.427	0.018
	anxiety	-0.306	0.211	-0.217	-1.450	0.152
	fear	-0.129	0.277	-0.060	-0.465	0.643
	gastrointestinal discomfort	1.153	0.737	0.186	1.566	0.123
	fatigue	0.089	0.177	0.065	0.505	0.615
	pruritus	0.249	0.325	0.085	0.767	0.446
	numbness	-0.572	0.947	-0.068	-0.604	0.548
	dizziness	-0.905	0.912	-0.113	-0.992	0.325
Day 4	pain	-0.303	0.260	-0.160	-1.162	0.250
	anxiety	0.123	0.326	0.054	0.377	0.708
	fear	-1.396	0.683	-0.254	-2.043	0.045
	gastrointestinal discomfort	-0.317	1.302	-0.043	-0.243	0.809
	fatigue	-0.218	0.198	-0.128	-1.100	0.276
	pruritus	-0.590	0.369	-0.182	-1.599	0.115
	numbness	-1.282	1.388	-0.171	-0.924	0.359
	dizziness	-3.490	1.853	-0.227	-1.884	0.064
Day 5	pain	-0.497	0.307	-0.197	-1.617	0.111
	anxiety	-0.030	0.470	-0.008	-0.063	0.950
	fear	-2.103	0.806	-0.297	-2.610	0.011
	gastrointestinal discomfort	-	-	-	-	-
	fatigue	0.026	0.308	0.010	0.083	0.934
	pruritus	-1.52	0.621	-0.280	-2.448	0.017
	numbness	-0.392	2.438	-0.018	-0.161	0.873
	dizziness	-	-	-	-	-

Table 7: Correlation between sleep efficiency and symptoms 5 days after trauma (n=69)

Time	Variable	Regression coefficient	Standard error	Standardized regression coefficient	*t*	*P*
Day 1	pain	-0.105	0.029	-0.466	-3.674	0.001
	anxiety	-0.001	0.018	-0.007	-0.054	0.957
	fear	-0.017	0.019	-0.116	-0.9	0.372
	gastrointestinal discomfort	-0.045	0.045	-0.108	-0.998	0.322

	fatigue	0.028	0.014	0.214	1.899	0.062
	pruritus	-0.03	0.038	-0.088	-0.782	0.437
	numbness	-0.046	0.027	-0.188	-1.732	0.088
	dizziness	0.014	0.032	0.049	0.443	0.659
Day 2	pain	-0.021	0.013	-0.209	-1.600	0.115
	anxiety	0	0.01	0.007	0.046	0.963
	fear	0.003	0.013	0.034	0.252	0.802
	gastrointestinal discomfort	0.034	0.04	0.097	0.832	0.408
	fatigue	0.014	0.008	0.220	1.703	0.094
	pruritus	-0.011	0.022	-0.057	-0.483	0.631
	numbness	-0.096	0.032	-0.36	-3.037	0.004
	dizziness	-0.011	0.029	-0.045	-0.364	0.717
Day 3	pain	-0.021	0.012	-0.266	-1.787	0.079
	anxiety	0.002	0.011	0.029	0.178	0.859
	fear	-0.002	0.015	-0.022	-0.155	0.878
	gastrointestinal discomfort	-0.022	0.039	-0.075	-0.571	0.570
	fatigue	0.008	0.009	0.113	0.807	0.423
	pruritus	-0.007	0.017	-0.048	-0.396	0.693
	numbness	-0.068	0.051	-0.166	-1.347	0.183
	dizziness	-0.014	0.049	-0.036	-0.286	0.776
Day 4	pain	-0.011	0.008	-0.094	-1.318	0.192
	anxiety	0.007	0.01	0.053	0.720	0.474
	fear	-0.047	0.022	-0.140	-2.175	0.034
	gastrointestinal discomfort	0.023	0.041	0.051	0.561	0.577
	fatigue	0.007	0.006	0.065	1.084	0.283
	pruritus	-0.158	0.012	-0.794	-13.483	＜0.001
	numbness	-0.191	0.044	-0.416	-4.347	＜0.001
	dizziness	-0.103	0.059	-0.109	-1.750	0.085
Day 5	pain	-0.019	0.012	-0.156	-1.551	0.126
	anxiety	0.006	0.018	0.032	0.299	0.766
	fear	0.012	0.033	0.036	0.377	0.708
	gastrointestinal discomfort	-	-	-	-	-
	fatigue	0.010	0.012	0.083	0.826	0.412
	pruritus	-0.179	0.024	-0.695	-7.343	＜0.001
	numbness	-0.053	0.096	-0.051	-0.548	0.586
	dizziness	-	-	-	-	-

Figures

FIG1 Sleep patterns 5 days after trauma

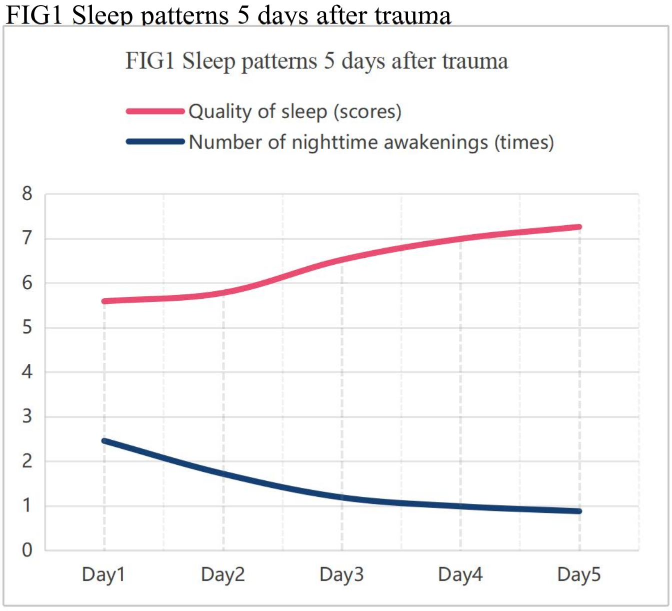

FIG2 Post-traumatic sleep-related symptom scores at 5 days

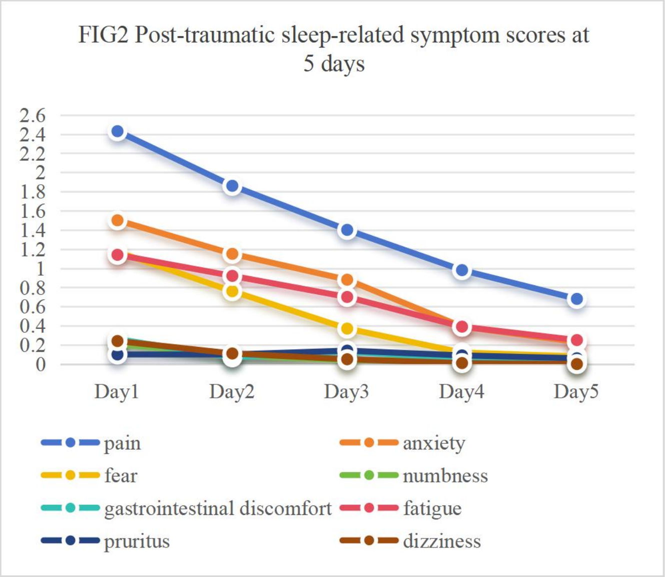

FIG3 Severity of sleep symptoms at different time periods during the first 5 days after trauma

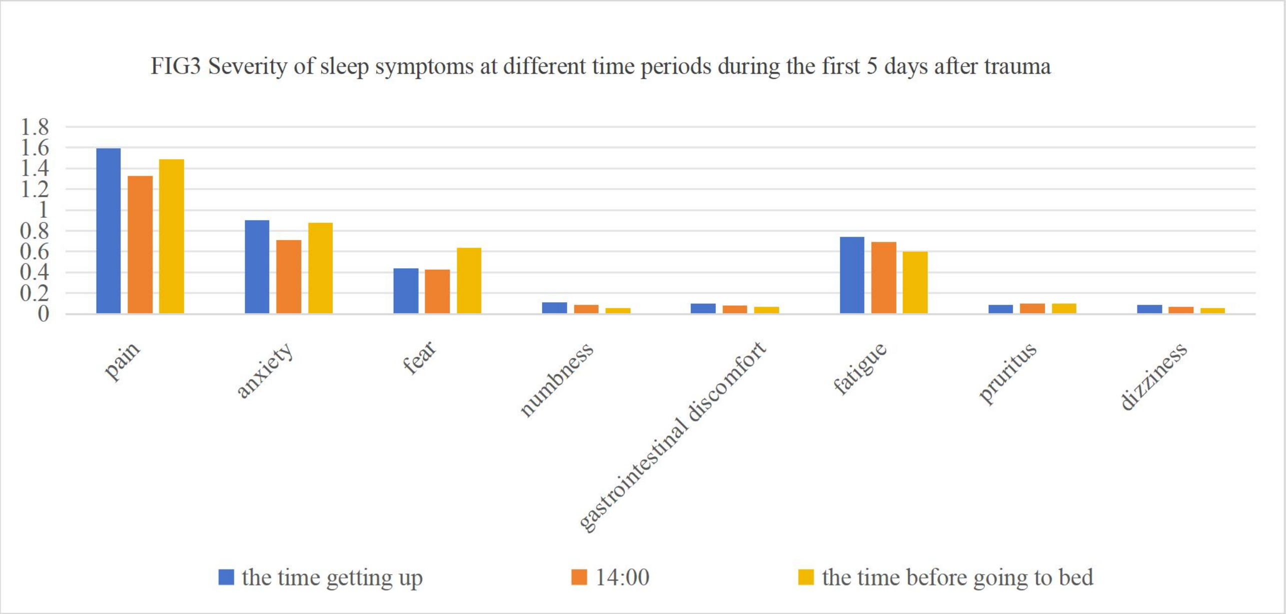

Supplement: Supplementary file 1 [file Datasheet1.pdf]
